# Sizing up beauty: Mechanisms of petal size regulation in ornamental plants

**DOI:** 10.1093/plphys/kiaf198

**Published:** 2025-05-22

**Authors:** Yunxiao Guan, Chui Eng Wong, Qiaoyu Zhang, Donghui Peng, Siren Lan, Fadi Chen, Zhong-Jian Liu, Hao Yu

**Affiliations:** Key Laboratory of National Forestry and Grassland Administration for Orchid Conservation and Utilization at College of Landscape Architecture and Art, Fujian Agriculture and Forestry University, Fuzhou 350002, China; Department of Biological Sciences, National University of Singapore, 14 Science Drive 4, 117543, Singapore; Department of Biological Sciences, National University of Singapore, 14 Science Drive 4, 117543, Singapore; Temasek Life Sciences Laboratory, 1 Research Link, National University of Singapore, 117604, Singapore; Key Laboratory of National Forestry and Grassland Administration for Orchid Conservation and Utilization at College of Landscape Architecture and Art, Fujian Agriculture and Forestry University, Fuzhou 350002, China; Key Laboratory of National Forestry and Grassland Administration for Orchid Conservation and Utilization at College of Landscape Architecture and Art, Fujian Agriculture and Forestry University, Fuzhou 350002, China; Key Laboratory of National Forestry and Grassland Administration for Orchid Conservation and Utilization at College of Landscape Architecture and Art, Fujian Agriculture and Forestry University, Fuzhou 350002, China; State Key Laboratory of Crop Genetics and Germplasm Enhancement, Key Laboratory of Landscaping, Ministry of Agriculture and Rural Affairs, College of Horticulture, Nanjing Agricultural University, Nanjing 210095, China; Key Laboratory of National Forestry and Grassland Administration for Orchid Conservation and Utilization at College of Landscape Architecture and Art, Fujian Agriculture and Forestry University, Fuzhou 350002, China; Department of Biological Sciences, National University of Singapore, 14 Science Drive 4, 117543, Singapore; Temasek Life Sciences Laboratory, 1 Research Link, National University of Singapore, 117604, Singapore

## Abstract

Ornamental plants can generate higher economic value per unit area compared with traditional crops. Enhancing market share in the flower industry relies on cultivating varieties with exceptional ornamental traits. Petal size, a critical factor influencing flower shape and ornamental appeal, is a primary focus for horticultural breeding selection. In this article, we review recent advances in understanding the regulation of petal size in ornamental plants through analyzing various patterns of cell division and expansion underlying petal growth and the genes involved in the related regulatory paradigms. We further highlight the intricate network of petal size control affected by multiple phytohormones and discuss several open questions and strategies for breeding ornamental plants with desired petal size traits based on current findings.

## Introduction

Flower size exhibits remarkable variation both among and within species, reflecting the diverse life strategies plants use to adapt to their environments. This diversity influences the evolutionary selection process by impacting reproduction (Davis et al. 2008; Endress 2011). In ornamental plants, flower size is crucial for attracting pollinators, such as bees, butterflies, and birds, thereby ensuring effective pollination essential for reproductive success and genetic diversity (Whitney et al. 2011; Lozada-Gobilard et al. 2023; Henríquez-Gangas et al. 2024). Furthermore, flower size affects shape and visual attractiveness, influencing marketability and consumer preference, as seen in the larger flowers of many cultivated floricultural plants compared with their wild counterparts. In angiosperms, the size of the perianth, particularly the petals, determines the overall flower size, making petal size a key trait for horticulturists.

**Figure 1.**
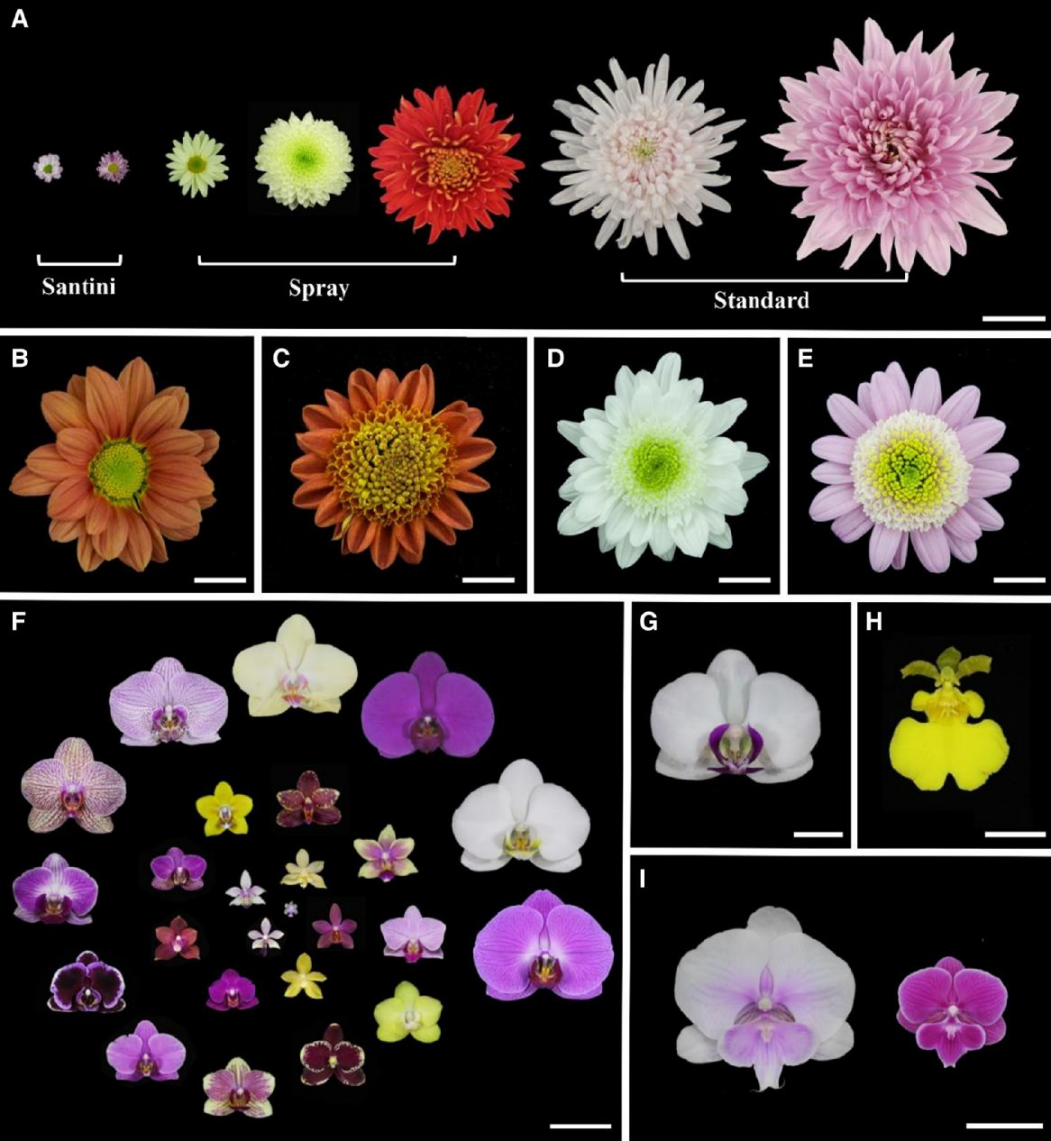
Diversity of petals and flowers in chrysanthemums and orchids. **A)** Three categories of chrysanthemum cut flowers. Scale bar: 4 cm. **B)** A typical chrysanthemum inflorescence (composite flower) consisting of marginal ray florets and inner whorls of disc florets. Scale bar: 2 cm. **C** to **E)** Three chrysanthemum varieties with anemone-type flowers. Both the ray floret petals and the disc florets that constitute prominent central hemispheres affect the composite flower diameter. Scale bars: 1 cm in C and 2 cm in **D** and **E)**. **F)** Different *Phalaenopsis* varieties with various petal and flower sizes. Scale bar: 5 cm. **G)** A typical *Phalaenopsis* flower. Scale bar: 2 cm. **H)** A typical *Oncidium* flower. Scale bar: 1 cm. **I)** Novel *Phalaenopsis* varieties with large lips. Scale bar: 4 cm.

For instance, petal size contributes considerably to the flower size and shape of the 2 largest families of flowering plants, Asteraceae and Orchidaceae. Chrysanthemums, 1 of the 4 major cut flowers in the world, constitute about 40 different species in the genus *Chrysanthemum* in the sunflower family Asteraceae. Chrysanthemum cut flowers can be divided into 3 categories partially based on flower size. The Santini category features flowers 1 to 3 cm in diameter, the Spray category includes flowers with diameters typically ranging from 4 to 10 cm, and the Standard category generally has larger flowers ranging from 11 to 20 cm in diameter (Fig. 1A). Like other Asteraceae plants, a typical chrysanthemum inflorescence (composite flower) comprises marginal ray florets and inner whorls of disc florets (Fig. 1B). The composite flower diameter is attributable to the number and pattern of both the ray floret petals and the disc florets that constitute prominent central hemispheres (Fig. 1, C to E).

A typical flower of orchids in the Orchidaceae family has 2 distinct types of petal structures, including 2 lateral petals adherent to the column that is characteristic of a unique merge of male and female organs and a specialized petal called the lip below the column. Variations in the size of lateral petals determine diverse flower diameters in some orchids, such as those in the *Phalaenopsis* genus (Fig. 1F). Furthermore, the relative sizes of lateral petals and lips also affect flower size and shape across orchid genera as exemplified by distinct flowers of *Phalaenopsis* and *Oncidium* genera. In *Phalaenopsis* flowers, lips are generally much smaller than lateral petals (Fig. 1G), whereas in *Oncidium* flowers, lips are relatively larger than lateral petals (Fig. 1H). In recent times, alteration of petal or lip size, as demonstrated by the large-lipped *Phalaenopsis* varieties (Fig. 1I), has been a popular approach to make flowers more visually striking and appealing, which is highly desirable in ornamental horticulture.

Overall, petal size is an important trait for enhancing the aesthetic appeal of ornamental plants. By manipulating petal size, horticulturists can develop new varieties with desirable traits, such as larger flower size or unique shape. Therefore, understanding the genetic and molecular mechanisms that control petal size is vital for breeding programs. In this review, we discuss recent advances in the understanding of petal size regulation pertaining to various patterns in cell division and expansion in ornamental plants and propose future research directions crucial for advancing the field and improving related breeding strategies.

## Petal growth pattern following primordium initiation

Petal development, like other lateral organs, progresses via a basic program comprising 3 crucial steps, including primordium initiation, cell proliferation, and cell expansion (Brioudes et al. 2009; Krizek and Anderson 2013; Huang and Irish 2015a). Most research on ornamental plants has so far focused on flower organ identity, with limited work on investigating the pattern of cell division and expansion underlying petal growth following primordium initiation, which, however, is a key factor affecting both final petal size and flower-opening characteristics (Sun et al. 2021).

**Figure 2.**
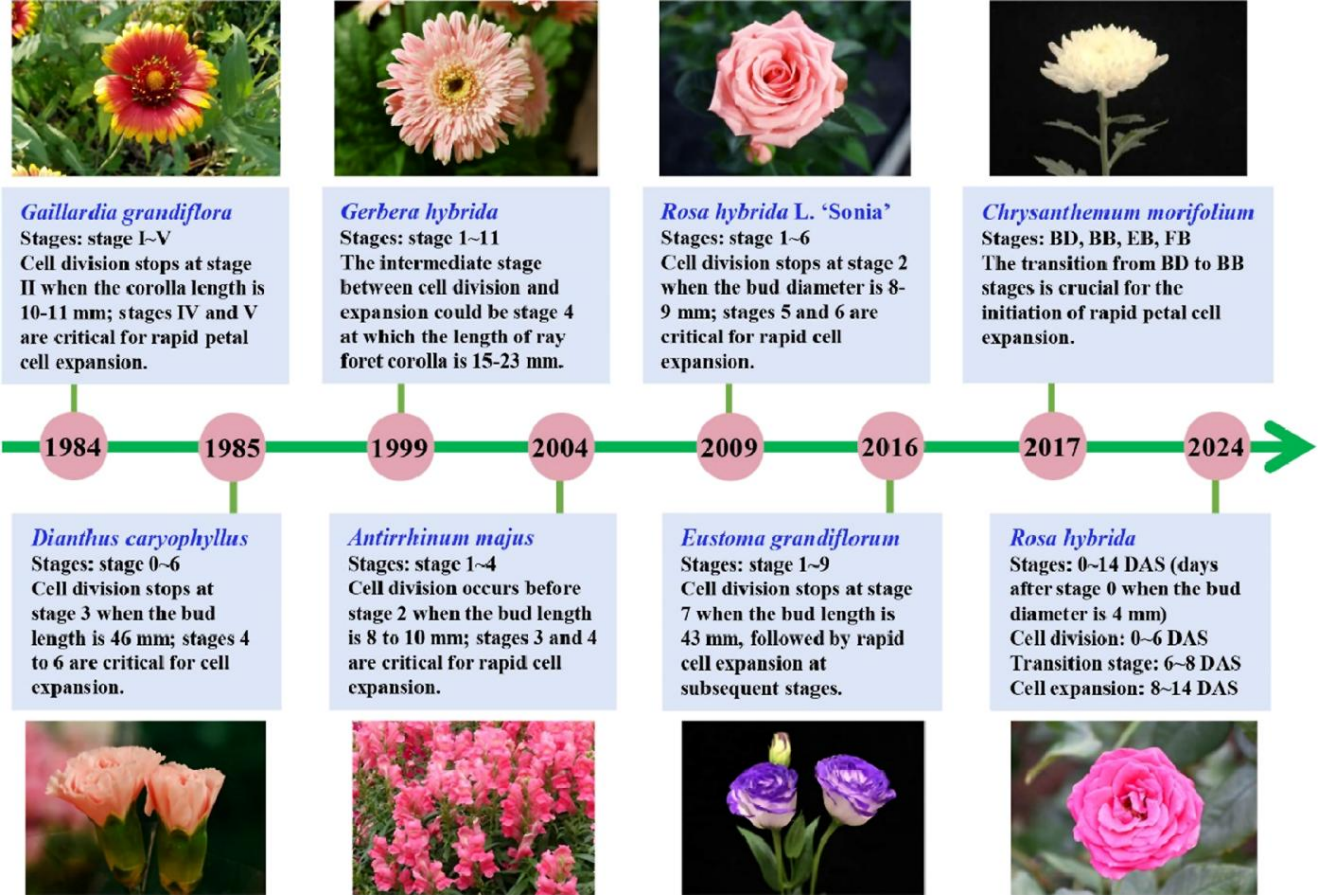
Progress in understanding patterns of cell division and expansion underlying petal growth during flower development in ornamental plants. Since 1984, investigation of cell division and expansion related to petal growth has been successively carried out in *Gaillardia grandiflora* Van Houtte ‘Goblin’ (Koning 1984), *Dianthus caryophyllus* L. ‘Sir Arthur’ (Kenis et al. 1985), *Gerbera hybrida ‘*Terra Regina’ (Kotilainen et al. 1999), *Antirrhinum majus* line 165E (Bey et al. 2004), *Rosa hybrida* L. ‘Sonia’ (Yamada et al. 2009), *Eustoma grandiflorum* ‘Asuka-no-Nami’ (Norikoshi et al. 2016), *Chrysanthemum morifolium ‘*Jinba’ (Wang et al. 2017), and *Rosa hybrida ‘*Samantha’ (Wang et al. 2024b). The temporal patterns of cell division and expansion during petal growth of each species are indicated in the figure. BD, budding stage; BB, bud breaking stage; EB, early blooming stage; FB, full blooming stage. For illustration purposes, the flower images here show varieties or cultivars in the same natural or artificial/hybrid species mentioned in the above references and may not be exact ones used in these studies.

To date, investigations of petal cell morphology (Box 1) associated with cell division and expansion have been carried out to understand petal growth patterns in several ornamental species (Fig. 2). Koning (1984) defined 5 stages for ray flower development in *Gaillardia grandiflora*, a hybrid species in the Asteraceae family, in which cell division in petals ceases at stage II when the corolla length reaches 10 to 11 mm. In contrast, cell expansion is predominant at stages IV and V, although it occurs throughout all 5 stages. In the carnation *Dianthus caryophyllus*, the petal growth process can be divided into 6 stages after the flower bud length reaches 1 cm. Measurements of petal dry weight and DNA content indicate that cell division ceases at stage 3, while petal growth at stages 4 to 6 primarily relies on cell expansion (Kenis et al. 1985). Microarray analysis of gene expression in petals at various stages of flower development in *Gerbera hybrida*, another species in the Asteraceae family, showed that stage 4 is the intermediate phase between cell division and expansion (Kotilainen et al. 1999; Laitinen et al. 2007). In the snapdragon *Antirrhinum majus*, Bey et al. (2004) divided the late petal development process into 4 distinct stages based on different floral bud lengths. Cell division occurs before stage 2 when the bud length reaches 8 to 10 mm, while cell expansion primarily contributes to petal growth at stages 3 and 4 (Rolland-Lagan et al. 2003; Bey et al. 2004). Yamada et al. (2009) examined petal cell morphology at all 6 stages of flower development of the rose *Rosa hybrida* L. ‘Sonia’ and observed that the number of abaxial epidermal cells increases until stage 2, while cell area continues to expand at all stages, particularly at stages 5 and 6.

Box 1. Petal anatomy Petal tissue typically comprises an adaxial and abaxial epidermis, with a central layer of parenchyma cells that house embedded vascular tissues. In most angiosperms, the adaxial epidermis contains conical-shaped cells, known as conical cells. These cells affect texture and tactile sensation, potentially aid in attracting pollinators and impacting color, wettability, light capture, and reflectance (Whitney et al. 2011). The abaxial epidermal cells are flatter and exhibit a wider range of shapes, from polygonal in *Phalaenopsis* (Hsieh et al. 2013) to rectangular in gerbera (Ren et al. 2018). Ma et al. (2008) identified 5 distinct sections in rose petals: adaxial epidermis, parenchyma cell, vascular bundle, abaxial subepidermis (AbsE), and abaxial epidermis. Among these, the AbsE consists of regularly shaped cells forming a flat layer, with their expansion closely linked to petal expansion. In chrysanthemum and gerbera, changes in overall petal size are measured by observing the number and size of AbsE cells (Ren et al. 2018; Guan et al. 2022). Nevertheless, in *Gaillardia grandiflora* and *Eustoma grandiflorum*, changes in petal size are assessed by measuring the cells at the adaxial epidermis (Koning 1984; Norikoshi et al. 2016).

In *Eustoma grandiflorum*, commonly known as lisianthus, petal development associated with increased length of floral buds from 4 to 46 mm was divided into 9 stages (Norikoshi et al. 2016). Examination of petal epidermal cells revealed that petal cell number stabilizes at stage 7, following which petals begin to rapidly expand. Wang et al. (2017) classified the inflorescence development of the chrysanthemum *Chrysanthemum morifolium* into 4 stages from budding (BD), bud breaking (BB), early blooming (EB), to full blooming (FB). RNA-seq analysis showed that the transition from BD to BB stages is crucial for initiating rapid petal elongation. More recently, Wang et al. (2024b) tracked changes in petal cell number and size in rose floral bud starting from a diameter of 4 mm and found that petal area increases slowly from 0 to 6 days after stage 0 (DAS), which is mainly driven by cell proliferation. A transition from cell proliferation to cell expansion occurs between 6 and 8 DAS, followed by rapid cell expansion from 8 to 14 DAS, resulting in a significant increase in petal area.

These results demonstrate that the 2 essential cellular processes, cell division and cell expansion (Box 2), determine the final cell number and area in petals, respectively, during flower development. Thus, analysis of the temporal patterns of cell division and expansion associated with petal morphology at different stages of flower development is essential for identifying key genes involved in these 2 processes, thereby facilitating the investigation of the regulatory mechanisms underlying petal size regulation.

Box 2. Cellular mechanics governing petal size Cell division contributes to petal growth by increasing the number of cells, while cell expansion allows post-mitotic cells to grow larger. This expansion is largely triggered by changes in turgor pressure, growth of the cell membrane and cell wall, and cytoskeleton remodeling (Pei et al. 2013). While early petal growth is driven by an increase in cytoplasmic mass, most of the increase in petal size results from post-mitotic cell expansion, primarily through vacuolar expansion (Wang et al. 2024b). Marker genes, such as cyclins (*CYCA1;1* and *CYCB1;2*) or *Proliferating cell nuclear antigen* (*PCNA*), are associated with cell division (Jin et al. 2025; Wang et al. 2024b). Meanwhile, genes like aquaporins (*PIP1;1* and *PIP2;1*), cellulose synthases (*CesA2* and *CesA3*), and expansins (*EXPA1*, *EXPA3*, and *EXPA8*) are crucial for cell expansion (Pei et al. 2013; Luo et al. 2013; Chen et al. 2013, 2020; Wang et al. 2024b). For instance, modifications in cell wall structure through expansins, a group of cell wall loosening enzymes, allow cells to grow in response to increased turgor pressure within the cell. Monitoring the expression of these genes alongside cell morphology can help to identify the specific timing of cell division and expansion, thereby facilitating the investigation into the regulatory mechanisms underlying petal size development.

### Petal cell proliferation

The timing of cell proliferation arrest in developing petals is one of the main factors affecting the final flower size. Extending the period when cells remain competent to divide can result in larger floral organs (Huang and Irish 2015a). In the model *Arabidopsis thaliana*, a series of genes has been identified that regulates petal size by influencing cell proliferation. For example, the AP2/ERF family genes *AINTEGUMENTA* (*ANT*) and *AINTEGUMENTA-LIKE 6* (*AIL6*), along with the C2H2 zinc finger transcription factor *JAGGED* (*JAG*), promote larger petals by prolonging the duration of cell division (Mizukami and Fischer 2000; Hu et al. 2003; Krizek 2009; Sauret-Güeto et al. 2013). In contrast, the ring-finger E3 ligase gene *BIG BROTHER* (*BB*), ubiquitin receptor gene *DA1*, CIN-like TCP family gene *TCP5*, and cyclin-dependent kinase (CDK) inhibitor genes *KIP RELATED PROTEIN 4* (*KRP4*) and *KRP2* negatively control petal size by inhibiting cell division (Disch et al. 2006; Li et al. 2008; Schiessl et al. 2014; Huang and Irish 2015b).

A group of genes that affect cell proliferation and petal size in ornamental plants has also been identified (Table 1). For example, various players related to the cytokinin pathway have been found to affect petal cell proliferation in rose (*Rose hybrida*). A R2R3-type MYB transcription repressor RhMYB73 recruits a co-repressor, RhTPL (TOPLESS homolog), and a histone deacetylase, RhHDA19, to form a repressor complex to downregulate the expression of a conserved microRNA *RhmiR159*, thereby accumulating the transcripts of its target gene *RhCKX6* that encodes a cytokinin oxidase affecting cytokinin catabolism. This leads to premature termination of petal cell division, ultimately resulting in smaller petals (Jing et al. 2023). Another transcription factor, RhMYC2, promotes cell proliferation by directly activating the cytokinin biosynthesis gene *RhLOG3*, thus positively regulating petal size (Gong et al. 2024). The cytokinin signaling protein RhARR14 activates the expression of an AP2/ERF transcription factor, *RhRAP2.4L*, and positively regulates cell proliferation by directly inhibiting the expression of the cell cycle inhibitor *KIP RELATED PROTEIN 2* (*RhKRP2*) (Wang et al. 2024b). As expected, silencing both *RhARR14* and *RhRAP2.4L* results in reduced petal size. In addition, the cytokinin response factor RhRR1 promotes petal cell division by elevating the expression of a GRAS family transcription factor, *RhSCL28*, which in turn upregulates the cyclin genes *RhCYCA1;1* and *RhCYCB1;2* (Jin et al. 2025).

**Table 1.** Genes affecting petal cell proliferation in ornamental plants.

Plant species	Genes	Function deduced from phenotypes	References
*Rose*	*RhmiR159*, *RhMYC2*, *RhLOG3*, *RhARR14*, *RhRAP2.4L*, *RhRR1*, *RhSCL28*, *RrANT1*	Promote cell proliferation	(Xu et al. 2022; Jing et al. 2023; Gong et al. 2024; Jin et al. 2025; Wang et al. 2024b)
	*RhCKX6*, *RhMYB73*	Inhibit cell proliferation	(Jing et al. 2023)
*Phalaenopsis*	*PebHLH*	Promote cell proliferation	(Hsieh et al. 2013)
	*PeMADS9*	Inhibit cell proliferation	(Hsieh et al. 2013)
*Oncidium*	*OAGL6-2*	Promote cell proliferation	(Chen et al. 2019)
*Chrysanthemum lavandulifolium*	*ClE2F1*	Promote cell proliferation	(Gao et al. 2018)
	*ClNAC84*, *ClMIP*	Inhibit cell proliferation	(Gao et al. 2022)
*Petunia hybrida*	*Grandiflora*	Promote cell proliferation	(Nishijima 2012)
*Antirrhinum majus*	*AmCIN*	Promote cell proliferation	(Crawford et al. 2004)
*Gerbera hybrida*	*GhCYC3*, *GhCYC4*	Promote cell proliferation	(Juntheikki-Palovaara et al. 2014)

In *Oncidium* orchids, the fully expanded lip is typically much larger than the petals in a mature flower (Fig. 1H). Chang et al. (2009) found that the cell size of the lip is similar to that of the petal, while the cell number is approximately 16 times greater. This indicates that the larger lip of *Oncidium* is primarily due to increased cell proliferation. A B-class MADS-box transcription factor, *OMADS5*, may negatively regulate lip formation by inhibiting petal cell division, as its expression is significantly reduced in lip-like petals and lip-like sepals (Chang et al. 2009). Virus-induced gene silencing of another MADS-box gene, *OAGL6-2*, in *Oncidium* and its ortholog, *PeMADS9*, in *Phalaenopsis* orchids results in opposite effects on the lip size by affecting cell proliferation, which is associated with reduced and increased expression of tetraspanin genes *OnAAF* and *PaAAF*, respectively (Chen et al. 2019). The related underlying mechanisms need to be further investigated. In addition, silencing of a bHLH transcription factor gene, *PebHLH*, also generates smaller flower size because of the premature termination of cell proliferation in *Phalaenopsis* (Hsieh et al. 2013).

In a wild chrysanthemum, *Chrysanthemum lavandulifolium*, overexpression of E2F transcription factor *ClE2F1* generates longer ligulate florets with an increased cell number (Gao et al. 2018). Moreover, Gao et al. (2022) found that the interaction between NAC transcription factor ClNAC84 and DUF59 protein ClMIP promotes the expression of a cyclin-dependent kinase gene, *ClKRP5*, to inhibit cell proliferation, resulting in smaller flowers. In petunia, the semi-dominant gene *Grandiflora* (*G*) plays a vital role in inducing large flower sizes, mainly by increasing cell numbers (Nishijima 2012). In *Antirrhinum*, the mutant of a TCP transcription factor gene *CINCINNATA*, *cin-755,* exhibits smaller petal lobes because of the reduced cell numbers (Crawford et al. 2004). In rose, an *AP2* gene *RrANT1* is abundantly transcribed in petals at the bud stage. The flower diameter of transgenic petunia overexpressing *RrANT* is increased by 1.2-fold compared with those in the wild-type background, which is attributable to an increased petal cell number (Xu et al. 2022). A similar function of *AmANT* has also been implicated in *Antirrhinum* (Kim et al. 2011). Additionally, overexpression of 2 TCP transcription factor genes, *GhCYC3* and *GhCYC4*, in gerbera (*Gerbera hybrida*) promotes cell division, resulting in longer trans and disc florets (Juntheikki-Palovaara et al. 2014).

### Petal cell expansion

When cells proliferate to a certain number, alterations in turgor pressure within the cell tissues, growth of the cell membrane and cell wall, and remodeling of the cytoskeleton are initiated, leading to rapid cell expansion (Pei et al. 2013). A few genes that influence floral organ size by affecting cell expansion have been identified in Arabidopsis. The most notable one is a bHLH transcription factor gene, *BIGPETALp* (*BPEp*), which is preferentially expressed in petals and negatively controls petal size by interfering with post-mitotic cell expansion (Szécsi et al. 2006). Given that cell expansion plays a predominant role in rapid petal expansion, this process not only determines petal size but also impacts the flower opening quality. Many genes associated with petal cell expansion have so far been identified in ornamental plants, particularly in roses, chrysanthemums, and gerberas (Table 2).

**Table 2.** Genes affecting petal cell expansion in ornamental plants.

Plant species	Genes	Function deduced from phenotypes	References
*Rosa hybrida*	*RhIAA14*, *RhNF-YC9*, *RhMYB6*, *RhPIP1;1*, *RhPIP2;1*, *RhBPEub*	Promote cell expansion	(Ma et al. 2008; Chen et al. 2013; Chen et al. 2020; Jia et al. 2022; Chen et al. 2023)
	*RhNAC100*, *RhGAI*, *RhARF2*, *RhRAP2.4L*	Inhibit cell expansion	(Luo et al. 2013; Pei et al. 2013; Chen et al. 2023; Wang et al. 2024b)
*Chrysanthemum morifolium*	*CmTCP20*, *CmBPE2*	Promote cell expansion	(Wang et al. 2019; Guan et al. 2022)
	*CmJAZ1-Like*, *CmGEG*	Inhibit cell expansion	(Guan et al. 2022; He et al. 2024)
*Gerbera hybrida*	*GhPRGL*, *Gh14-3-3b*, *Gh14-3-3f*	Promote cell elongation	(Lin et al. 2021; Jiang et al. 2022)
	*GhMIF, GhGEG*, *GhEIL1*, *GhWIP2*, *GhTCP7*, *GhIAA26*, *GhBPE*	Inhibit cell expansion	(Kotilainen et al. 1999; Han et al. 2017; Ren et al. 2018; Huang et al. 2020; Jiang et al. 2022; Ren et al. 2023)
*Lilium longiflorum*	*LS6K1*	Inhibit cell expansion	(Tzeng et al. 2009)
*Petunia hybrida*	*PhDof28*	Promote cell expansion	(Yue et al. 2023)
*Phalaenopsis*	*PeMADS1*	Promote cell expansion	(Hsieh et al. 2013)

Several genes have been reported to positively affect petal cell expansion in rose, including an Aux/IAA family gene, *RhIAA14*, a NF-YC transcription factor gene, *RhNF-YC9*, a R2R3 MYB transcription factor gene, *RhMYB6*, and 2 aquaporin genes, *RhPIP1;1*, and *RhPIP2;1*, as transiently silencing them causes diminished petal size (Ma et al. 2008; Chen et al. 2013, 2020, 2023; Jia et al. 2022). Conversely, another NAC transcription factor, *RhNAC100*, the DELLA gene *RhGAI*, and the auxin response factor gene *RhARF2* negatively control petal size by repressing cell expansion (Luo et al. 2013; Pei et al. 2013; Chen et al. 2023). In addition to affecting petal cell proliferation, RhRAP2.4L also limits petal cell expansion by directly inhibiting the expression of a bHLH gene, *BIG PETALub* (*RhBPEub*) (Wang et al. 2024b). Overall, almost all the genes mentioned above are shown to modulate rose petal size by regulating cell expansion-related genes, such as α-expansin (EXPA) protein genes (*RhEXPA1*, *RhEXPA4*, and *RhEXPA8*), xyloglucan endotransglycosylase/hydrolase (XTH) gene (*RhXTH6*), aquaporins genes (*RhPIP1;1* and *RhPIP2;1*), and cellulose synthase (CES) genes (*RhCesA2* and *RhCesA3*). These genes can serve as markers for assessing the changes in cell expansion in rose.

In chrysanthemum, a TCP family gene *CmTCP20* is abundantly transcribed in ray florets, and transgenic plants overexpressing *CmTCP20* exhibits larger flower diameter and ray petal size attributed to the elongated cell length (Wang et al. 2019). Inversely, a TIFY family protein CmJAZ1-like negatively controls ray petal size by interacting with a bHLH transcription factor, CmBPE2, a positive regulator of petal cell expansion, to attenuate its activation on the expansin-related gene *CmEXPA7* (Guan et al. 2022). He et al. (2024) identified a gibberellic acid (GA) stimulated in Arabidopsis (GASA) gene, *CmGEG*, in chrysanthemum ‘Jinba’ which is highly expressed in ray florets and functions in repressing petal cell elongation. In gerbera, the suppressors of petal expansion so far identified include a mini zinc-finger gene, *GhMIF*; an ethylene insensitive 3-like 1 gene, *GhEIL1*; a GASA gene, *GhGEG*; a WIP zinc finger protein gene, *GhWIP2*; a TCP family gene, *GhTCP7*; an auxin-responsive gene, *GhIAA26*; and a bHLH gene, *GhBPE* (Kotilainen et al. 1999; Han et al. 2017; Ren et al. 2018, 2023; Jiang et al. 2022). These genes limit ray petal size by inhibiting cell expansion. On the contrary, another GASA gene, *GhPRGL* and two 14-3-3 family genes, *Gh14-3-3b* and *Gh14-3-3f*, positively regulate ray petal length by promoting cell elongation (Lin et al. 2021; Jiang et al. 2022).

Furthermore, ectopic expression of the p70^s6k^ gene *LS6K1*, isolated from lily (*Lilium longiflorum*), in Arabidopsis results in shorter petals and stamens because of reduced cell size (Tzeng et al. 2009). In petunia, overexpression of a DNA-binding 1 finger (Dof)-type transcription factor *PhDof28* shows increased flower diameters and longer corolla tubes as a result of expanded cell size (Yue et al. 2023). In *Phalaenopsis* orchid, silencing a C-class MADS-box gene, *PeMADS1*, results in smaller flowers, a phenotype primarily caused by reduced petal cell expansion (Hsieh et al. 2013).

Although most reported genes function in either petal cell proliferation or petal cell expansion, some regulatory factors can participate in both processes. For example, RhRAP2.4L plays a dual role in rose petal growth, promoting cell proliferation at early stages while inhibiting cell expansion at later stages (Wang et al. 2024b). This suggests that the same genes may engage different interacting partners or downstream targets to separately affect cell proliferation and expansion. In addition, functional conservation and divergence of orthologous genes, such as *BPEp* orthologs, are also observable in petal size regulation in different plant species. *RhBPEub* in rose and *CmBPE2* in chrysanthemum promote petal cell expansion, whereas *GhBPE* in gerbera negatively regulates petal size by inhibiting cell elongation (Guan et al. 2022; Jiang et al. 2022; Wang et al. 2024b). Similarly, *OAGL6-2* in *Oncidium* and its ortholog *PeMADS9* in *Phalaenopsis* exhibit opposite functions in affecting cell proliferation and lip size (Chen et al. 2019). Thus, critical outstanding questions include addressing the molecular basis and dynamics of functional conservation and divergence among these orthologous genes in petal size regulation.

## Phytohormones-mediated regulation of petal size

**Figure 3.**
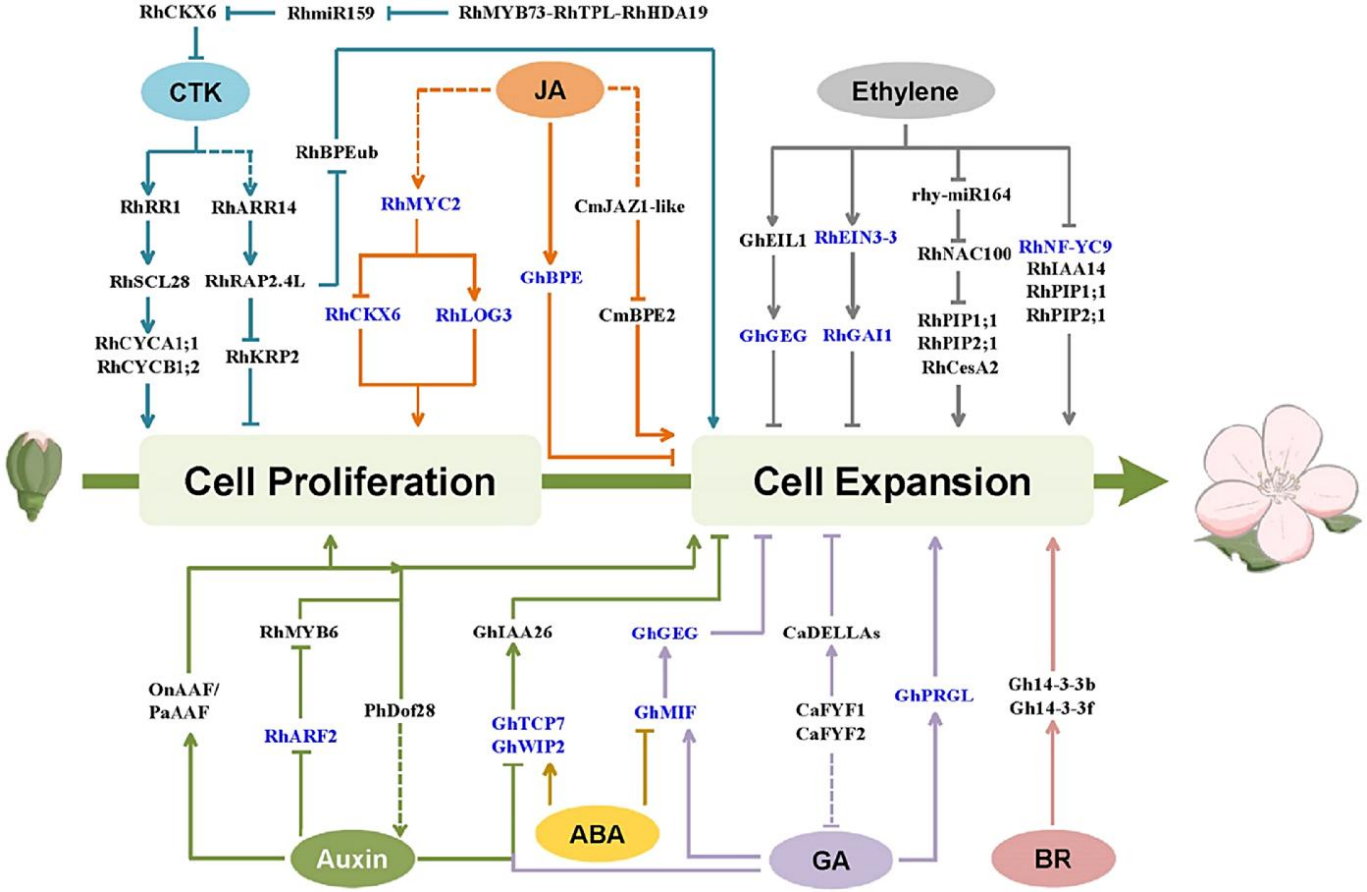
Hormone-mediated regulatory network of petal growth in ornamental plants. Petal growth from a floral bud to a fully open flower comprises 2 main successive processes, cell proliferation and cell expansion. Pathways related to the action of 7 phytohormones are represented by different colors. Arrows and bars indicate positive and negative regulations, respectively. Dashed lines represent predicted regulatory effects. Genes involved in hormone crosstalk are highlighted in blue. *Rh*, *Rosa hybrida*; *Gh*, *Gerbera hybrida*; *Cm*, *Chrysanthemum morifolium*; *On*, *Oncidium*; *Pa*, *Phalaenopsis*; *Ph*, *Petunia hybrida*; *Ca*, *Cattleya*.

Phytohormones play essential roles in regulating various processes of plant growth and development. Given that the petal is the key and showy floral organ of ornamental plants, the effects of phytohormones on petal growth have garnered considerable attention. As summarized below, the phytohormones, such as auxin, ethylene, GA, cytokinin (CTK), abscisic acid (ABA), jasmonic acid (JA), and brassinolide (BR), have all been implicated in regulating petal size through modulating cell proliferation and/or cell expansion in a complex regulatory network (Fig. 3).

### Auxin

Exogenous application of IBA to chrysanthemum petals at the early blooming stage results in longer petals, with significant increases in both cell number throughout the petals and cell length in the basal region (Wang et al. 2017). In addition, RNA-seq analysis has revealed that auxin-related genes were markedly differentially expressed across 4 stages of petal elongation. In *Oncidium* and *Phalaenopsis* orchids, Auxin Activation Factor (AAF) homologs are positively associated with petal and lip size by regulating cell division and expansion (Chen et al. 2019). In rose, petal expansion is promoted under NAA treatment, which downregulates the auxin response factor gene *RhARF2* that represses petal cell expansion by inhibiting *RhMYB6* expression. Interestingly, the ethylene signaling genes *RhETR3* and *RhEIN3* are significantly reduced in the *RhARF2*-silenced petals compared with the control, indicating that RhARF2 may control petal size by mediating the crosstalk between auxin and ethylene signaling (Chen et al. 2023) (Fig. 3).

In petunia, overexpression of *PhDof28* increases the endogenous IAA levels and triggers the expression of cell expansion-related genes, thereby promoting petal expansion (Yue et al. 2023). In gerbera, 2 transcription factors, GhWIP2 and GhTCP7, form a heterodimer to activate the auxin signal repressor gene *GhIAA26*, leading to a repression on petal expansion. This regulatory process is involved in the crosstalk of ABA, GA, and auxin (Ren et al. 2018, 2023) (Fig. 3). GA and auxin inhibit the transcription of *GhWIP2* and *GhTCP7* to positively control petal expansion, whereas ABA activates the expression of *GhWIP2* to restrict petal expansion. Overall, these results demonstrate that auxin and its crosstalk with other phytohormone pathways positively regulate petal size.

### Ethylene

The effects of ethylene on petal growth during flower opening and their underlying regulatory mechanisms have been studied extensively in rose. Ethylene treatment significantly reduces petal width and length, whereas 1-Methylcyclopropene (1-MCP), a competitive inhibitor of ethylene action, promotes petal elongation primarily by regulating cell expansion (Ma et al. 2008). A group of genes that are responsive to ethylene to affect petal expansion have been identified. The expression of *RhPIP1;1*, *RhPIP2;1*, *RhNF-YC9*, and *RhIAA14* is repressed by ethylene, and transient silencing of these genes leads to reduced petal expansion and smaller flower size (Ma et al. 2008; Chen et al. 2013, 2020; Jia et al. 2022). Moreover, knockdown of the NF-YC transcription factor gene *RhNF-YC9* reduces the expression of a GA biosynthetic gene, *RhGA20ox*, but significantly elevates the transcript level of a GA catabolic gene, *RhGA2ox*, decreasing the content of GA_4_ and GA_7_. These results suggest that *RhNF-YC9* may affect petal expansion through mediating the crosstalk between ethylene and GA (Chen et al. 2020) (Fig. 3). This crosstalk is also supported by another observation where a DELLA gene, *RhGAI1*, is induced by RhEIN3-3, a primary regulator in ethylene signaling, and thereby inhibits petal size by regulating the expression of genes involved in cell expansion (Zhao and Guo 2011; Luo et al. 2013). In addition, ethylene attenuates *rhy-miR164* abundance, thus reducing its cleavage of *RhNAC100* and indirectly inhibiting the expression of *RhPIP1;1*, *RhPIP2;1*, and *RhCesA2*, which results in smaller petals (Pei et al. 2013).

Similarly, induction of ethylene response by 1-aminocyclopropane-1-carboxylic acid treatment significantly inhibits petal growth in gerbera, while those treated with 1-MCP exhibit longer petals. In addition, an *ETHYLENE INSENSITIVE 3-Like 1* gene in gerbera, *GhEIL1*, is induced by 1-aminocyclopropane-1-carboxylic acid and inhibits ray petal elongation by activating *GhGEG*, a gerbera homolog of the gibberellin-stimulated transcript 1 from tomato (Huang et al. 2020). Taken together, ethylene limits petal size primarily by restricting cell expansion.

### Cytokinin

Cytokinin plays pivotal roles in plant organ development by regulating cell division (Sakakibara 2006; Werner and Schmülling 2009). In rose, cytokinin levels in petals decline during early flower development, coinciding with decreased cell proliferation (Jing et al. 2023). Exogenous application of 6-benzylaminopurine (6-BA) before 5 DAS prolongs cell division and enlarges petals, while 6-BA treatment at 8 DAS reduces petal size because of decreased cell size (Wang et al. 2024b). These findings indicate that the timing of cytokinin application significantly impacts petal growth. Moreover, the RhMYB73-RhTPL-RhHDA19 repression complex promotes *RhCKX6* expression, resulting in premature cytokinin clearance and subsequent reduction in petal size (Jing et al. 2023).

The cytokinin signaling protein RhARR14 promotes the expression of *RhRAP2.4L*, which enhances cell proliferation while inhibiting cell expansion by repressing the expression of *RhKRP2* and *RhBPEub*, respectively, thereby regulating petal size (Wang et al. 2024b). Additionally, the cytokinin response factor RhRR1 stimulates *RhSCL28* expression to promote cell division via upregulating *RhCYCA1;1* and *RhCYCB1;2* during early stages of petal growth (Jin et al. 2025). Overall, cytokinin plays a dual role in determining petal size by promoting cell proliferation, but inhibiting cell expansion, in the course of petal development.

### Gibberellin

In gerbera, GA positively regulates petal growth by promoting petal elongation (Zhang et al. 2012; Li et al. 2015). *GhPRGL*, a GASA family gene, is stimulated by GA, and promotes petal elongation at early petal growth stages in gerbera. Meanwhile, GhBPE induced by JA directly inhibits the expression of *GhPRGL* to limit petal elongation at late stages. The coordinated regulation of JA and GA ensures that petals develop with an appropriate size (Jiang et al. 2022). Furthermore, the GhMIF-*GhGEG* module negatively regulates the corolla length of ray florets by mediating the crosstalk between GA and ABA, with their expression induced by GA_3_ but inhibited by ABA (Kotilainen et al. 1999; Han et al. 2017) (Fig. 3). In addition, ectopic expression of 2 MADS-box genes, *CaFYF1* and *CaFYF2*, identified from *Cattleya intermedia*, a native species of Cattleya orchids, in Arabidopsis causes shorter sepals and petals through negative regulation of GA response by elevating the expression of *DELLA* genes (Chen et al. 2018). These observations, together with GA effect on *GhWIP2* expression and RhNF-YC9 role in influencing GA levels (Fig. 3), suggest that the GA signaling pathway interacts with various phytohormone pathways to control petal cell expansion.

### Other hormones

Methyl jasmonate treatment induces the expression of *RhMYC2*, which is a crucial component of the JA signaling pathway and controls petal size by integrating cytokinin and JA signaling in rose (Gong et al. 2024). At early petal growth stages, high JA levels promote *RhMYC2* expression, which in turn activates a cytokinin biosynthetic gene *RhLOG3* and inhibits the cytokinin oxidase gene *RhCKX6* to sustain elevated cytokinin levels (Fig. 3). This synergistic action stimulates cell division and positively impacts petal size. In contrast, at later growth stages, declining JA levels lead to cytokinin degradation, resulting in the cessation of cell division. In gerbera, JA inhibits cell elongation of ray petals by promoting the expression of *GhBPE* (Jiang et al. 2022), while CmJAZ1-like protein, a repressor of the JA signaling pathway, restricts ray petal cell expansion of chrysanthemum by repressing the transcriptional activity of CmBPE2 (Guan et al. 2022). These results demonstrate a dual role of JA in controlling petal size through affecting both cell proliferation and expansion.

ABA treatment results in shorter petals and antagonizes GA to regulate cell expansion during petal growth in gerbera (Li et al. 2015). 14-3-3 proteins are the crucial regulators of the BR signaling pathway (Gampala et al. 2007), and their homologs in gerbera, Gh14-3-3b and Gh14-3-3f, affect cell elongation and petal length, indicating a role of BR in affecting petal size (Lin et al. 2021).

In summary, auxin, GA, cytokinin, and BR generally promote petal growth during flower opening, whereas ethylene, JA, and ABA exert the opposite effects. In addition, the effects of these phytohormones on petal size display the following 3 features. First, some hormones, such as cytokinin and JA, trigger temporally specific and dose-dependent responses during petal development. Second, a phytohormone may switch its role in promoting and restricting petal size in the course of petal development. For example, although GA generally promotes petal elongation, it may activate inhibitory factors as a feedback mechanism to prevent excessive petal growth (Han et al. 2017). Lastly, there is sophisticated crosstalk among these phytohormones, including interactions between JA and cytokinin, ethylene and GA, GA and JA, GA and ABA, GA and auxin, and ethylene and auxin (Li et al. 2015; Ren et al. 2018, 2023; Chen et al. 2020; Jia et al. 2022; Jiang et al. 2022; Gong et al. 2024). These interactions permit plants to respond to various signals to modulate petal size by affecting a wide range of genes related to both cell proliferation and expansion.

## Effects of environmental factors on petal size

Petal growth and development are not only regulated by genetic factors but are also influenced by various environmental factors. So far, a few studies have demonstrated that environmental factors, such as light and temperature, can directly affect petal size, although the underlying mechanisms remain unclear. In *Gerbera hybrida*, light signaling significantly promotes petal length and width by enhancing cell expansion (Zhang et al. 2012). A genome-wide association study based on 295 rapeseed accessions with variations in petal size has identified a transcription factor, Far-Red Elongated Hypocotyl 3 (FHY3), that regulates petal size in the light signaling pathway (Wang et al. 2024a). Furthermore, CRISPR-Cas9–mediated knockout of *BnFHY3* leads to a reduction in petal size, suggesting that the photochrome-mediated light signaling pathway may be involved in the regulation of rapeseed petal size. Another study has shown that the inflorescence diameter of the herbaceous peony slightly increases under shade (Zhao et al. 2012). In addition to light, elevated temperature promotes the accumulation of endogenous ABA in petunia petals, thereby inhibiting cell division in developing flower buds (Sood et al. 2021). The molecular basis of the regulatory effects of these external conditions on petal size needs to be further explored.

## Conclusions and future directions

Abundant variation in flower size is crucial for evolutionary divergence by altering reproductive barriers and mating systems (Sargent et al. 2007; Brunet 2009; Goodwillie et al. 2009; Schiestl and Schlüter 2009). Beyond its evolutionary significance, flower size is a key trait in ornamental plants, directly impacting their aesthetic and commercial value (Fig. 1). Large flowers create a striking visual appeal, while small flowers contribute to texture and contrast in landscape designs.

Since petal size primarily determines flower diameter, it is critical to understand petal growth patterns by examining their morphological changes during flower opening. However, such research remains limited to a few ornamental species (Fig. 2). Recent studies integrating morphological analyses with transcriptomics have identified key genes controlling petal cell proliferation and expansion, some of which have been functionally characterized (Tables 1 and 2). These findings have led to a preliminary regulatory network for petal size control, emphasizing the critical role of phytohormones in coordinating cell division and expansion (Fig. 3). Despite these advances, our understanding of petal size regulation is still in its infancy, with many open questions remaining.

One key challenge is deciphering how environmental factors influence the molecular framework of petal size regulation, as petal size is a quantitative trait affected by external conditions (Zhang et al. 2012; Martínez-Díaz et al. 2024). Additionally, the dynamic interplay between regulatory factors controlling cell proliferation and expansion at different developmental stages remains largely unexplored. A deeper understanding of the spatial and temporal regulation of these processes is crucial for elucidating the molecular basis of petal size determination. While current studies have focused primarily on ethylene, GA, cytokinin, and auxin, future research should also investigate the roles of JA, BR, and ABA, as well as their interactions in modulating petal size.

To enhance petal size traits in ornamental plants, we propose the following breeding approaches based on multifaced mechanisms and genetic resources explored so far. First, breeding programs can develop new varieties with larger flowers and unique shapes by crossbreeding with exotic germplasm from wild relatives or underutilized species. Second, natural and induced mutations through chemical or physical mutagenesis have been successfully employed to develop unique ornamental traits and could similarly be applied to modify petal size (Okamura et al. 2014; Soliman et al. 2014; Su et al. 2019; Din et al. 2023). Third, ploidy manipulation, achieved through methods like colchicine treatment and somatic cell hybridization, offers a promising approach for generating polyploid plants with enhanced vigor and larger flowers (Ollitrault et al. 2020; Touchell et al. 2020; Kosser et al. 2022). Fourth, petal size can be controlled by applying phytohormones, though optimizing timing and dosage of treatment is essential for specific varieties and growth conditions to achieve the desired outcome. Lastly, advanced techniques, such as CRISPR-Cas9, have been applied in many ornamental plants (Tang et al. 2023). Petal size could be manipulated through editing genes involved in cell division and expansion.

By integrating these approaches, ornamental plant breeders can develop flowers with optimized aesthetics and market appeal. Future research should focus on elucidating the comprehensive regulatory network of petal size control and bridging the gap between fundamental studies and practical applications in horticulture. A deeper understanding of petal growth dynamics will enable the development of a more diverse array of ornamental plants with varying sizes and captivating floral forms, enriching both scientific knowledge and the floriculture industry.

Advances box

- Petal size is primarily determined by the interplay between initial cell proliferation and subsequent cell expansion.
- Most genes involved in petal size regulation affect either cell proliferation or cell expansion, although some of them have been shown to influence both processes.
- Phytohormones modulate petal growth dynamics, with GA, cytokinin, and BR generally promoting petal growth, while ethylene, JA, and ABA exert the opposite effect.
- Different phytohormones interact cooperatively or antagonistically to regulate petal size by influencing genes related to cell proliferation and expansion.

Outstanding questions box

- What are the specific downstream targets of key transcription factors, such as TCPs, bHLHs, ARFs, and AP2/ERF? How do these networks interact to balance petal growth and pattern formation?
- How are different phytohormonal signals integrated temporally and spatially to regulate cell division and expansion in petals?
- How do environmental factors affect genetic and hormonal pathways to influence petal size?
- How do epigenetic and epitranscriptomic modifications influence the expression of genes involved in petal size control?
- What causes the formation of different types of petals, such as lips and petals in orchids, and distinct petals of ray florets, trans florets, and disc florets in Asteraceae? What are the main factors responsible for the difference in their sizes?

## Data Availability

The data underlying this article are available in the manuscript.

## Dive Curated Terms

The following phenotypic, genotypic, and functional terms are of significance to the work described in this paper:

FHY3 Gramene: AT3G22170
FHY3 Araport: AT3G22170

KRP2 Gramene: AT3G50630
KRP2 Araport: AT3G50630

KRP4 Gramene: AT2G32710
KRP4 Araport: AT2G32710

tcp5 Gramene: AT5G60970
tcp5 Araport: AT5G60970

AIL6 Gramene: AT5G10510
AIL6 Araport: AT5G10510

CESA2 Gramene: AT4G39350
CESA2 Araport: AT4G39350

petal AmiGo: PO:0009032

AP2 Gramene: AT4G36920
AP2 Araport: AT4G36920

CYCA1;1 Gramene: AT1G44110
CYCA1;1 Araport: AT1G44110

## References

[kiaf198-B1] Bey M, Stüber K, Fellenberg K, Schwarz-Sommer Z, Sommer H, Saedler H, Zachgo S. Characterization of antirrhinum petal development and identification of target genes of the class B MADS box gene *DEFICIENS*. Plant Cell. 2004:16(12):3197–3215. 10.1105/tpc.104.026724 15539471 PMC535868

[kiaf198-B2] Brioudes F, Joly C, Szécsi J, Varaud E, Leroux J, Bellvert F, Bertrand C, Bendahmane M. Jasmonate controls late development stages of petal growth in *Arabidopsis thaliana*. Plant J. 2009:60(6):1070–1080. 10.1111/j.1365-313X.2009.04023.x 19765234

[kiaf198-B3] Brunet J . Pollinators of the rocky mountain columbine: temporal variation, functional groups and associations with floral traits. Ann Bot. 2009:103(9):1567–1578. 10.1093/aob/mcp096 19414518 PMC2701757

[kiaf198-B4] Chang Y-Y, Kao N-H, Li J-Y, Hsu W-H, Liang Y-L, Wu J-W, Yang C-H. Characterization of the possible roles for B class MADS box genes in regulation of perianth formation in orchid. Plant Physiol. 2009:152(2):837–853. 10.1104/pp.109.147116 20018605 PMC2815905

[kiaf198-B5] Chen C, Hussain N, Ma Y, Zuo L, Jiang Y, Sun X, Gao J. The ARF2-MYB6 module mediates auxin-regulated petal expansion in rose. J Exp Bot. 2023:74(15):4489–4502. 10.1093/jxb/erad173 37158672

[kiaf198-B6] Chen C, Hussain N, Wang Y, Li M, Liu L, Qin M, Ma N, Gao J, Sun X. An ethylene-inhibited NF-YC transcription factor RhNF-YC9 regulates petal expansion in rose. Hortic Plant J. 2020:6(6):419–427. 10.1016/j.hpj.2020.11.007

[kiaf198-B7] Chen W, Yin X, Wang L, Tian J, Yang R, Liu D, Yu Z, Ma N, Gao J. Involvement of rose aquaporin RhPIP1;1 in ethylene-regulated petal expansion through interaction with RhPIP2;1. Plant Mol Biol. 2013:83(3):219–233. 10.1007/s11103-013-0084-6 23748738

[kiaf198-B8] Chen WH, Hsu WH, Hsu HF, Yang CH. A tetraspanin gene regulating auxin response and affecting orchid perianth size and various plant developmental processes. Plant Direct. 2019:3(8):e00157. 10.1002/pld3.157 31406958 PMC6680136

[kiaf198-B9] Chen W-H, Lee Y-I, Yang C-H. Ectopic expression of two *FOREVER YOUNG FLOWER* orthologues from *Cattleya* orchid suppresses ethylene signaling and DELLA results in delayed flower senescence/abscission and reduced flower organ elongation in *Arabidopsis*. Plant Mol Biol Rep. 2018:36(5–6):710–724. 10.1007/s11105-018-1114-y

[kiaf198-B10] Crawford B, Nath U, Carpenter R, Coen E. *CINCINNATA* controls both cell differentiation and growth in petal lobes and leaves of Antirrhinum. Plant Physiol. 2004:135(1):244–253. 10.1104/pp.103.036368 15122032 PMC429364

[kiaf198-B11] Davis C, Endress P, Baum D. Evolution of floral gigantism. Curr Opin Plant Biol. 2008:11(1):49–57. 10.1016/j.pbi.2007.11.003 18207449

[kiaf198-B12] Din A, Qadri ZA, Wani M, Banday N, Iqbal S, Nazki I, Wani F. Enhancing flower color diversity in *Chrysanthemum* cv. “Candid” through ethyl methane sulfonate mutagenesis: a promising approach for ornamental crop improvement. ACS Agric Sci Technol. 2023:3(1):1004–1013. 10.1021/acsagscitech.3c00200

[kiaf198-B13] Disch S, Anastasiou E, Sharma VK, Laux T, Fletcher JC, Lenhard M. The E3 ubiquitin ligase BIG BROTHER controls *Arabidopsis* organ size in a dosage-dependent manner. Curr Biol. 2006:16(3):272–279. 10.1016/j.cub.2005.12.026 16461280

[kiaf198-B14] Endress PK . Evolutionary diversification of the flowers in angiosperms. Am J Bot. 2011:98(3):370–396. 10.3732/ajb.1000299 21613132

[kiaf198-B15] Gampala S, Kim T-W, He J-X, Tang W, Deng Z, Bai M, Guan S, Lalonde S, Sun Y, Gendron J, et al An essential role for 14-3-3 proteins in brassinosteroid signal transduction in *Arabidopsis*. Dev Cell. 2007:13(2):177–189. 10.1016/j.devcel.2007.06.009 17681130 PMC2000337

[kiaf198-B16] Gao R, Wang H, Qi X, Zhu L, Yang X, Chen S, Jiang J, Wang Z, Chen F. ClNAC84 interacts with ClMIP to regulate the cell cycle and reduce the size of *Chrysanthemum lavandulifolium* organs. Front Hortic. 2022:1:1042105. 10.3389/fhort.2022.1042105

[kiaf198-B17] Gao R, Yan Y, Yang X, Wang Y, Fang W, Chen S, Jiang J, Wang H, Chen F. Cle2f1 overexpression enhances plant growth in *Chrysanthemum lavandulifolium* (Fisch. ex Trautv.) makino. Plant Mol Biol Rep. 2018:36(2):341–349. 10.1007/s11105-018-1084-0

[kiaf198-B18] Gong F, Jing W, Jin W, Liu H, Zhang Y, Wang R, Wei Y, Tang K, Jiang Y, Gao J, et al RhMYC2 controls petal size through synergistic regulation of jasmonic acid and cytokinin signaling in rose. Plant J. 2024:120(2):459–472. 10.1111/tpj.16993 39164914

[kiaf198-B19] Goodwillie C, Sargent R, Eckert C, Elle E, Geber M, Johnston M, Kalisz S, Moeller D, Ree R, Vallejo-Marin M, et al Correlated evolution of mating system and floral display traits in flowering plants and its implications for the distribution of mating system variation. New Phytol. 2009:185(1):311–321. 10.1111/j.1469-8137.2009.03043.x 19807872

[kiaf198-B20] Guan Y, Ding L, Jiang J, Jia D, Li S, Jin L, Zhao W, Zhang X, Song A, Chen S, et al The TIFY family protein CmJAZ1-like negatively regulates petal size via interaction with the bHLH transcription factor CmBPE2 in *Chrysanthemum morifolium*. Plant J. 2022:112(6):1489–1506. 10.1111/tpj.16031 36377371

[kiaf198-B21] Han M, Jin X, Yao W, Kong L, Huang G, Tao Y, Li L, Wang X, Wang Y. A Mini zinc-finger protein (MIF) from *Gerbera hybrida* activates the GASA protein family gene, *GEG*, to inhibit ray petal elongation. Front Plant Sci. 2017:8:1649. 10.3389/fpls.2017.01649 29018462 PMC5615213

[kiaf198-B22] He Z, Jiang R, Wang X, Wang Y. A GASA protein family gene, *CmGEG*, inhibits petal growth in Chrysanthemum. Int J Mol Sci. 2024:25(6):3367. 10.3390/ijms25063367 38542341 PMC10970651

[kiaf198-B23] Henríquez-Gangas F, Reyes S, Carvallo G. Flower size constrains reproductive potential in three Andean monkeyflower species. Plant Spec Biol. 2024:39(1):26–38. 10.1111/1442-1984.12442

[kiaf198-B24] Hsieh MH, Pan ZJ, Lai PH, Lu HC, Yeh HH, Hsu CC, Wu WL, Chung MC, Wang SS, Chen WH, et al Virus-induced gene silencing unravels multiple transcription factors involved in floral growth and development in Phalaenopsis orchids. J Exp Bot. 2013:64(12):3869–3884. 10.1093/jxb/ert218 23956416 PMC3745740

[kiaf198-B25] Hu Y, Xie Q, Chua NH. The Arabidopsis auxin-inducible gene ARGOS controls lateral organ size. Plant Cell. 2003:15(9):1951–1961. 10.1105/tpc.013557 12953103 PMC181323

[kiaf198-B26] Huang G, Han M, Jian L, Chen Y, Sun S, Wang X, Wang Y. An ETHYLENE INSENSITIVE3-LIKE1 protein directly targets the *GEG* promoter and mediates ethylene-induced ray petal elongation in *Gerbera hybrida*. Front Plant Sci. 2020:10:1737. 10.3389/fpls.2019.01737 32038696 PMC6993041

[kiaf198-B27] Huang T, Irish VF. Gene networks controlling petal organogenesis. J Exp Bot. 2015a:67(1):61–68. 10.1093/jxb/erv444 26428062

[kiaf198-B28] Huang T, Irish VF. Temporal control of plant organ growth by TCP transcription factors. Curr Biol. 2015b:25(13):1765–1770. 10.1016/j.cub.2015.05.024 26073137

[kiaf198-B29] Jia Y, Chen C, Gong F, Jin W, Zhang H, Qu S, Ma N, Jiang Y, Gao J, Sun X. An Aux/IAA family member, *RhIAA14*, involved in ethylene-inhibited petal expansion in Rose (*Rosa hybrida*). Genes (Basel). 2022:13(6):1041. 10.3390/genes13061041 35741802 PMC9222917

[kiaf198-B30] Jiang R, Yuan W, Yao W, Jin X, Wang X, Wang Y. A regulatory GhBPE-GhPRGL module maintains ray petal length in *Gerbera hybrida*. Mol Hortic. 2022:2(1):9. 10.1186/s43897-022-00030-3 37789358 PMC10515009

[kiaf198-B31] Jin W, Gong F, Zhang Y, Wang R, Liu H, Wei Y, Tang K, Jiang Y, Gao J, Sun X. Cytokinin-responsive RhRR1-RhSCL28 transcription factor module positively regulates petal size by promoting cell division in rose. J Exp Bot. 2025:76(2):381–392. 10.1093/jxb/erae331 39230685

[kiaf198-B32] Jing W, Gong F, Liu G, Deng Y, Liu J, Yang W, Sun X, Li Y, Gao J, Zhou X, et al Petal size is controlled by the MYB73/TPL/HDA19-miR159-CKX6 module regulating cytokinin catabolism in Rosa hybrida. Nat Commun. 2023:14(1):7106. 10.1038/s41467-023-42914-y 37925502 PMC10625627

[kiaf198-B33] Juntheikki-Palovaara I, Tähtiharju S, Lan T, Broholm SK, Rijpkema AS, Ruonala R, Kale L, Albert VA, Teeri TH, Elomaa P. Functional diversification of duplicated CYC2 clade genes in regulation of inflorescence development in *Gerbera hybrida* (Asteraceae). Plant J. 2014:79(5):783–796. 10.1111/tpj.12583 24923429

[kiaf198-B34] Kenis J, Silvente S, Trippi V. Nitrogen metabolism and senescence associated changes during growth of carnation flowers (Dianthus caryophyllus). Physiol Plantarum. 1985:65(4):455–459. 10.1111/j.1399-3054.1985.tb08673.x

[kiaf198-B35] Kim BM, Inaba J-i, Masuta C. Virus induced gene silencing in *Antirrhinum majus* using the *Cucumber mosaic* virus vector: functional analysis of the AINTEGUMENTA (Am-ANT) gene of *A. majus*. Hortic Environ Biote. 2011:52(2):176–182. 10.1007/s13580-011-0172-y

[kiaf198-B36] Koning R . The roles of plant hormones in the growth of the corolla of Gaillardia Grandiflora (Asteraceae) ray flowers. Am J Bot.1984:71(1):1–8. 10.1002/j.1537-2197.1984.tb12478.x

[kiaf198-B37] Kosser S, Bakshi P, Bhat DJ, Jasrotia A, Angmo T, Nisa Q. Ploidy manipulation in fruit crops: a review. Indian J of Hill Farming. 2022:35:119–136. 10.56678/iahf-2022.35.02.17

[kiaf198-B38] Kotilainen M, Helariutta Y, Mehto M, Pollanen E, Albert VA, Elomaa P, Teeri TH. *GEG* participates in the regulation of cell and organ shape during corolla and carpel development in *Gerbera hybrida*. Plant Cell. 1999:11(6):1093–1104. 10.1105/tpc.11.6.1093 10368180 PMC144246

[kiaf198-B39] Krizek B . *AINTEGUMENTA* and *AINTEGUMENTA-LIKE6* act redundantly to regulate Arabidopsis floral growth and patterning. Plant Physiol. 2009:150(4):1916–1929. 10.1104/pp.109.141119 19542297 PMC2719149

[kiaf198-B40] Krizek BA, Anderson JT. Control of flower size. J Exp Bot. 2013:64(6):1427–1437. 10.1093/jxb/ert025 23404902

[kiaf198-B41] Laitinen R, Pöllänen E, Teeri T, Elomaa P, Kotilainen M. Transcriptional analysis of petal organogenesis in *Gerbera hybrida*. Planta. 2007:226:347–360. 10.1007/s00425-007-0486-2 17334783

[kiaf198-B42] Li L, Zhang W, Zhang L, Li N, Peng J, Wang Y, Zhong C, Yang Y, Sun S, Liang S, et al Transcriptomic insights into antagonistic effects of gibberellin and abscisic acid on petal growth in *Gerbera hybrida*. Front Plant Sci. 2015:6:168. 10.3389/fpls.2015.00168 25852718 PMC4362084

[kiaf198-B43] Li Y, Zheng L, Corke F, Smith C, Bevan MW. Control of final seed and organ size by the DA1 gene family in *Arabidopsis thaliana*. Genes Dev. 2008:22(10):1331–1336. 10.1101/gad.463608 18483219 PMC2377187

[kiaf198-B44] Lin X, Huang S, Huang G, Chen Y, Wang X, Wang Y. 14-3-3 proteins are involved in BR-induced ray petal elongation in *Gerbera hybrida*. Front Plant Sci. 2021:12:718091. 10.3389/fpls.2021.718091 34421972 PMC8371339

[kiaf198-B45] Lozada-Gobilard S, Nielsen N, Sapir Y. Flower size as an honest signal in royal irises (*Iris* section *Oncocyclus*, Iridaceae). Plants (Basel, Switzerland). 2023:12(16):2978. 10.3390/plants12162978 37631189 PMC10459770

[kiaf198-B46] Luo J, Ma N, Pei H, Chen J, Li J, Gao J. A *DELLA* gene, *RhGAI1*, is a direct target of EIN3 and mediates ethylene-regulated rose petal cell expansion via repressing the expression of *RhCesA2*. J Exp Bot. 2013:64(16):5075–5084. 10.1093/jxb/ert296 24014864 PMC3830487

[kiaf198-B47] Ma N, Xue J, Li Y, Liu X, Dai F, Jia W, Luo Y, Gao J. *Rh-PIP2;1*, a rose aquaporin gene, is involved in ethylene-regulated petal expansion. Plant Physiol. 2008:148(2):894–907. 10.1104/pp.108.120154 18715962 PMC2556823

[kiaf198-B48] Martínez-Díaz Y, Espinosa-García FJ, Martén-Rodríguez S, García-Rodríguez YM, Cuevas E. Floral attractants in an alpine environment: linking floral volatiles, flower size and pollinators. Alpine Bot. 2024:134(1):101–114. 10.1007/s00035-023-00303-7

[kiaf198-B49] Mizukami Y, Fischer RL. Plant organ size control: *AINTEGUMENTA* regulates growth and cell numbers during organogenesis. Proc Natl Acad Sci U S A. 2000:97(2):942–947. 10.1073/pnas.97.2.942 10639184 PMC15435

[kiaf198-B50] Nishijima T . Large flower size: molecular basis and role of cytokinin. J Jpn Soc Hortic Sci. 2012:81(2):129–139. 10.2503/jjshs1.81.129

[kiaf198-B51] Norikoshi R, Shibata T, Ichimura K. Cell division and expansion in petals during flower development and opening in *Eustoma grandiflorum*. Horticult J. 2016:85(2):154–160. 10.2503/hortj.MI-071

[kiaf198-B52] Okamura M, Hase Y, Furusawa Y, Tanaka A. Tissue-dependent somaclonal mutation frequencies and spectra enhanced by ion beam irradiation in chrysanthemum. Euphytica. 2014:202(3):333–343. 10.1007/s10681-014-1220-3

[kiaf198-B53] Ollitrault P, Germanà MA, Froelicher Y, Cuenca J, Aleza P, Morillon R, Grosser J, Guo W. Ploidy manipulation for citrus breeding, genetics, and genomics. In: Gentile A, La Malfa S, Deng Z, editors. The citrus genome genome. Springer Nature; 2020. p. 75–105. 10.1007/978-3-030-15308-3_6

[kiaf198-B54] Pei H, Ma N, Tian J, Luo J, Chen J, Li J, Zheng Y, Chen X, Fei Z, Gao J. An NAC transcription factor controls ethylene-regulated cell expansion in flower petals. Plant Physiol. 2013:163(2):775–791. 10.1104/pp.113.223388 23933991 PMC3793057

[kiaf198-B55] Ren G, Li L, Huang Y, Wang Y, Zhang W, Zheng R, Zhong C, Wang X. GhWIP2, a WIP zinc finger protein, suppresses cell expansion in *Gerbera hybrida* by mediating crosstalk between gibberellin, abscisic acid, and auxin. New Phytol. 2018:219(2):728–742. 10.1111/nph.15175 29681133

[kiaf198-B56] Ren G, Li L, Patra B, Li N, Zhou Y, Zhong C, Wang Y, Yuan L, Wang X. The transcription factor GhTCP7 suppresses petal expansion by interacting with the WIP-type zinc finger protein GhWIP2 in *Gerbera hybrida*. J Exp Bot. 2023:74(14):4093–4109. 10.1093/jxb/erad152 37102769

[kiaf198-B57] Rolland-Lagan AG, Bangham JA, Coen E. Growth dynamics underlying petal shape and asymmetry. Nature. 2003:422(6928):161–163. 10.1038/nature01443 12634785

[kiaf198-B58] Sakakibara H . Cytokinins: activity, biosynthesis, and translocation. Annu Rev Plant Biol. 2006:57(1):431–449. 10.1146/annurev.arplant.57.032905.105231 16669769

[kiaf198-B59] Sargent R, Goodwillie C, Kalisz S, Ree RH. Phylogenetic evidence for a flower size and number trade-off. Am J Bot. 2007:94(12):2059–2062. 10.3732/ajb.94.12.2059 21636399

[kiaf198-B60] Sauret-Güeto S, Schiessl K, Bangham A, Sablowski R, Coen E. *JAGGED* controls *Arabidopsis* petal growth and shape by interacting with a divergent polarity field. PLoS biol. 2013:11(4):e1001550. 10.1371/journal.pbio.1001550 23653565 PMC3641185

[kiaf198-B61] Schiessl K, Muiño JM, Sablowski R. Arabidopsis JAGGED links floral organ patterning to tissue growth by repressing kip-related cell cycle inhibitors. Proc Natl Acad Sci U S A. 2014:111(7):2830–2835. 10.1073/pnas.1320457111 24497510 PMC3932912

[kiaf198-B62] Schiestl FP, Schlüter PM. Floral isolation, specialized pollination, and pollinator behavior in orchids. Annu Rev Entomol. 2009:54(1):425–446. 10.1146/annurev.ento.54.110807.090603 19067636

[kiaf198-B63] Soliman T, Lv S, Yang H, Hong B, Ma N, Zhao L. Isolation of flower color and shape mutations by gamma radiation of *Chrysanthemum morifolium* Ramat cv. Youka. Euphytica. 2014:199(3):317–324. 10.1007/s10681-014-1127-z

[kiaf198-B64] Sood A, Duchin S, Adamov Z, Carmeli-Weissberg M, Shaya F, Spitzer-Rimon B. Abscisic acid mediates the reduction of petunia flower size at elevated temperatures due to reduced cell division. Planta. 2021:255(1):18. 10.1007/s00425-021-03807-w 34894276

[kiaf198-B65] Su J, Jiang J, Zhang F, Liu Y, Ding L, Chen S, Chen F. Current achievements and future prospects in the genetic breeding of chrysanthemum: a review. Hortic Res. 2019:6(1):109. 10.1038/s41438-019-0193-8 31666962 PMC6804895

[kiaf198-B66] Sun X, Qin M, Yu Q, Huang Z, Xiao Y, Li Y, Ma N, Gao J. Molecular understanding of postharvest flower opening and senescence. Mol Hortic. 2021:1(1):7. 10.1186/s43897-021-00015-8 37789453 PMC10514961

[kiaf198-B67] Szécsi J, Joly C, Bordji K, Varaud E, Cock JM, Dumas C, Bendahmane M. *BIGPETALp*, a *bHLH* transcription factor is involved in the control of *Arabidopsis* petal size. EMBO J. 2006:25(16):3912–3920. 10.1038/sj.emboj.7601270 16902407 PMC1553195

[kiaf198-B68] Tang J, Ye J, Liu P, Wang S, Chen F, Song A. Ornamental plant gene editing: past, present and future. Ornam Plant Res. 2023:3(1):1–6. 10.48130/OPR-2023-0006

[kiaf198-B69] Touchell D, Palmer I, Ranney T. *In vitro* ploidy manipulation for crop improvement. Front Plant Sci. 2020:11:722. 10.3389/fpls.2020.00722 32582252 PMC7284393

[kiaf198-B70] Tzeng TY, Kong LR, Chen CH, Shaw CC, Yang CH. Overexpression of the lily p70(s6k) gene in Arabidopsis affects elongation of flower organs and indicates TOR-dependent regulation of AP3, PI and SUP translation. Plant Cell Physiol. 2009:50(9):1695–1709. 10.1093/pcp/pcp114 19651701

[kiaf198-B71] Wang J, Guan Y, Ding L, Li P, Zhao W, Jiang J, Chen S, Chen F. The *CmTCP20* gene regulates petal elongation growth in *Chrysanthemum morifolium*. Plant Sci. 2019:280:248–257. 10.1016/j.plantsci.2018.12.008 30824003

[kiaf198-B72] Wang J, Wang H, Ding L, Song A, Shen F, Jiang J, Chen S, Chen F. Transcriptomic and hormone analyses reveal mechanisms underlying petal elongation in *Chrysanthemum morifolium* ‘Jinba’. Plant Mol Biol. 2017:93(6):593–606. 10.1007/s11103-017-0584-x 28108965

[kiaf198-B73] Wang R, Li Y, Xu S, Huang Q, Tu M, Zhu Y, Cen H, Dong J, Jiang L, Yao X. Genome-wide association study reveals the genetic basis for petal-size formation in rapeseed (*Brassica napus*) and CRISPR-Cas9-mediated mutagenesis of BnFHY3 for petal-size reduction. Plant J. 2024a:118(2):373–387. 10.1111/tpj.16609 38159103

[kiaf198-B74] Wang Y, Qin M, Zhang G, Lu J, Zhang C, Ma N, Sun X, Gao J. Transcription factor RhRAP2.4L orchestrates cell proliferation and expansion to control petal size in rose. Plant Physiol. 2024b:194(4):2338–2353. 10.1093/plphys/kiad657 38084893

[kiaf198-B75] Werner T, Schmülling T. Cytokinin action in plant development. Curr Opin Plant Biol. 2009:12(5):527–538. 10.1016/j.pbi.2009.07.002 19740698

[kiaf198-B76] Whitney H, Bennett K, Dorling M, Sandbach L, Prince D, Chittka L, Glover B. Why do so many petals have conical epidermal cells? Ann Bot. 2011:108(4):609–616. 10.1093/aob/mcr065 21470973 PMC3170151

[kiaf198-B77] Xu Y, Xing Y, Wei T, Wang P, Liang Y, Xu M, Ding H, Wang J, Feng L. Transcription factor *RrANT1* of *Rosa rugosa* positively regulates flower organ size in *Petunia hybrid*. Int J Mol Sci. 2022:23(3):1236. 10.3390/ijms23031236 35163160 PMC8835453

[kiaf198-B78] Yamada K, Norikoshi R, Suzuki K, Nishijima T, Imanishi H, Ichimura K. Cell division and expansion growth during rose petal development. J Jpn Soc Hortic Sci. 2009:78(3):356–362. 10.2503/jjshs1.78.356

[kiaf198-B79] Yue Y, Zhu W, Shen H, Wang H, Du J, Wang L, Hu H. DNA-binding one finger transcription factor *PhDof28* regulates petal size in Petunia. Int J Mol Sci. 2023:24(15):11999. 10.3390/ijms241511999 37569375 PMC10418906

[kiaf198-B80] Zhang L, Li L, Wu J, Peng J, Zhang L, Wang X. Cell expansion and microtubule behavior in ray floret petals of *Gerbera hybrida*: responses to light and gibberellic acid. Photochem Photobiol Sci. 2012:11(2):279–288. 10.1039/c1pp05218g 22020373

[kiaf198-B81] Zhao D, Hao Z, Tao J. Effects of shade on plant growth and flower quality in the herbaceous peony (*Paeonia lactiflora Pall*). Plant Physiol Bioch. 2012:61:187–196. 10.1016/j.plaphy.2012.10.005 23141672

[kiaf198-B82] Zhao Q, Guo H-W. Paradigms and paradox in the ethylene signaling pathway and interaction network. Mol Plant. 2011:4(4):626–634. 10.1093/mp/ssr042 21690206

